# Survey dataset on architect׳s awareness and adoption of building envelope technologies for energy efficient housing in Lagos State

**DOI:** 10.1016/j.dib.2018.06.093

**Published:** 2018-07-03

**Authors:** Adedotun O. Akinola, Albert B. Adeboye, Adedapo Oluwatayo, Oluwole Alagbe, Oluwatosin Babalola, Adedeji O. Afolabi

**Affiliations:** a Department of Architecture, Covenant University, Nigeria; b Department of Building Technology, Covenant University, Nigeria

**Keywords:** Architect, Building envelope technologies, Construction industry, Energy efficiency, Housing

## Abstract

Low energy houses are forms of housing that use less energy from the design, technologies and building products from any source than a traditional or average contemporary house. The survey dataset examines architect׳s awareness and adoption of building envelope technologies (BET) for energy efficient housing in Lagos State, Nigeria. The dataset was based on seventy-four (74) returned questionnaires of both registered and non-registered Architects. A multistage sampling that involved cluster sampling and random sampling of architects in Lagos State was adopted. Descriptive statistical tools were used to present the dataset. The dataset contains the intent of promoting energy sustainability by architect while designing their building envelopes, the awareness of the building envelope strategies to adopt, factors influencing their adoption of these strategies, strategies that can be adopted to improve adoption of building envelope technologies for energy efficiency in housing units. The dataset can be used for evolving housing energy policy by decision makers.

**Specifications Table**

**Table:** 

Subject area	*Environmental Sciences*
More specific subject area	*Building envelope technologies*
Type of data	*Tables and Figures*
How data was acquired	*Field Survey*
Data format	*Raw and analyzed*
Experimental factors	*Cross-sectional survey research design of registered and non-registered Architects*
Experimental features	*Cluster and random sampling selection, charts and tables*
Data source location	*Lagos, Nigeria*
Data accessibility	*All the data are in this data article*

**Value of the data**

• In most developing countries, there are few empirical evidence on the adoption of building envelope technologies which can be used by manufacturers in order to produce better energy efficient technologies for housing and to develop housing energy codes.
• Due to issues of climate change, sustainability and environmental pollution there is need for decision makers, researchers and construction professionals to enact and implement energy efficiency policies which would be convenient to occupants and positively impact the environment.
• A lot of energy is being generated for housing consumption, there is need to create sustainable solutions through designs which can be attained through innovative building envelopes that takes cognizance of low energy consumption and produce effective thermal comfort.
• Prospective and current architects involved in housing designs can benefit from the dataset through the variables that indicate building envelope technologies that can help attain energy efficient housing units.
• The dataset can be beneficial to developing countries that have problems in power generation by ensuring that buildings are habitable in spite of the non-availability of energy at any time of the day especially in hot dry regions.
• Based on the human behavioural studies on perception of architects, the dataset can be replicated in other developing countries to understand the designers’ take on the use of building envelope technologies and how it can be attained through different design strategies.

## 1 Data

**Fig. 1:**
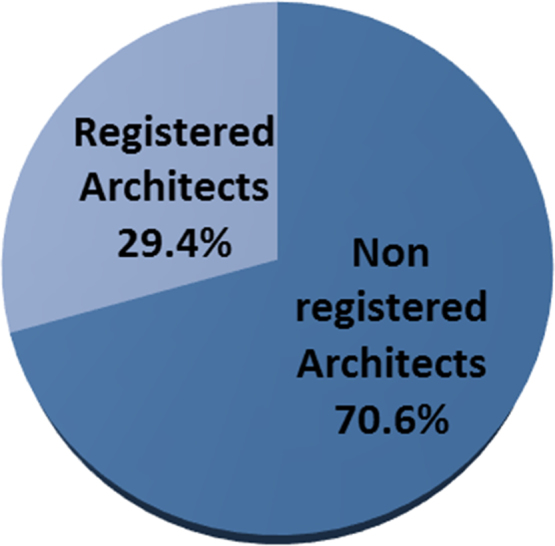
Registration status.

**Fig. 2:**
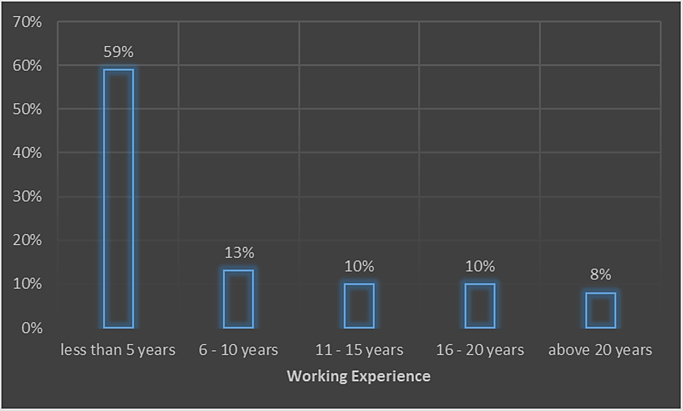
Industry working experience.

**Fig. 3:**
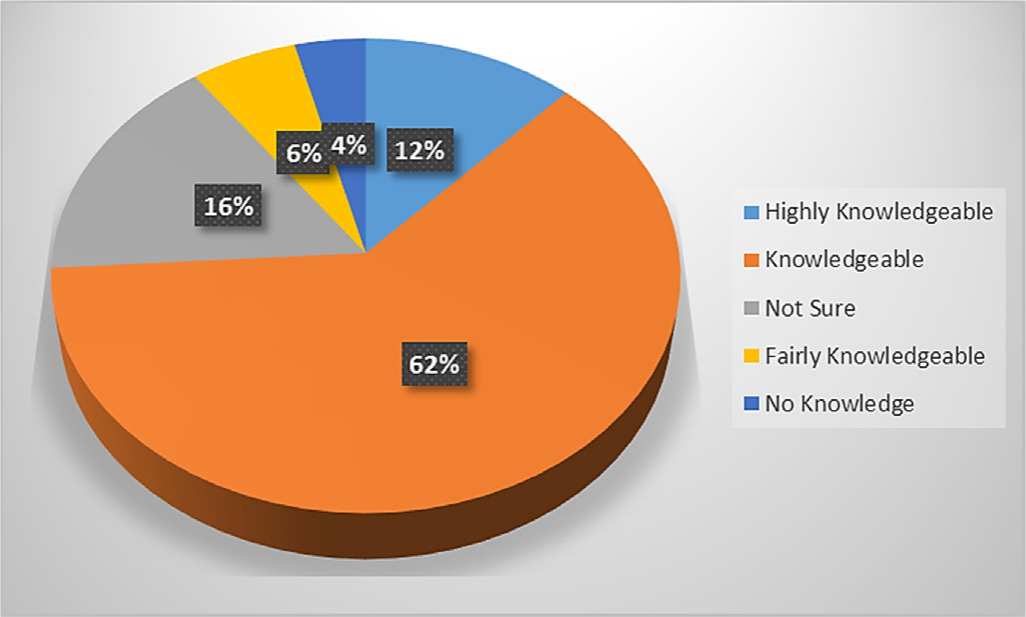
Knowledge of building envelope technologies.

**Fig. 4:**
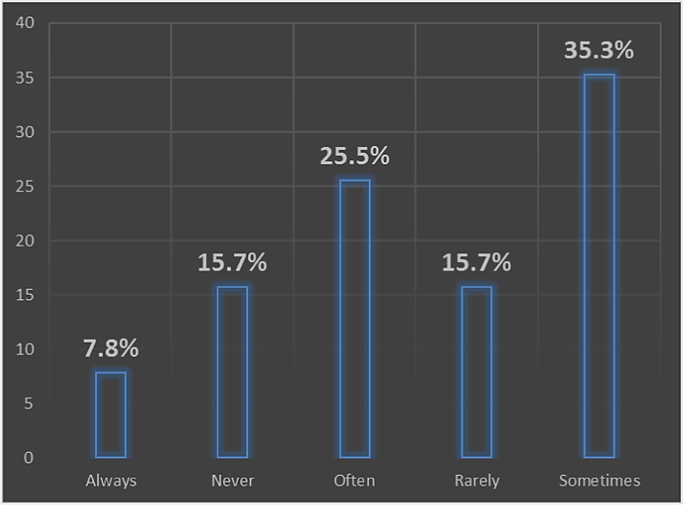
Intention to promote building envelope technologies in energy efficient housing units.

**Fig. 5:**
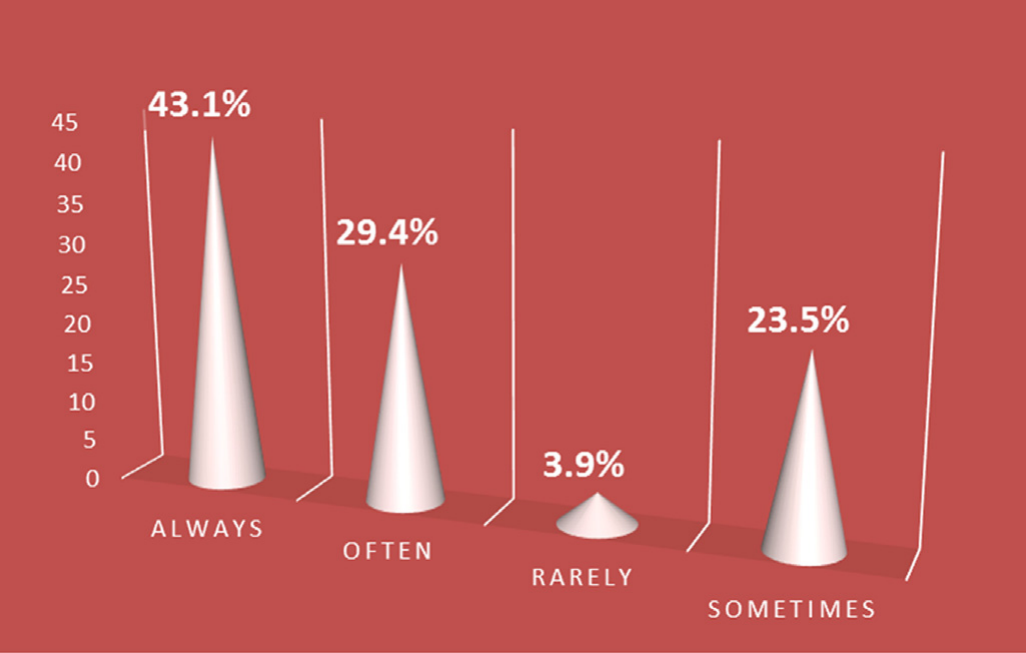
Reduction of energy consumption using building envelope technologies.

A properly designed and constructed building envelope can greatly increase a building׳s energy savings, comfort, and indoor air quality. Building envelope technologies can reduce uncontrolled air and moisture exchange, decrease thermal losses and gains, and improve occupant comfort [1], [2], [3], [4]. The development of responsive/dynamic building envelope strategies and technologies, adapting to transient external and internal boundary conditions, is considered a crucial step towards the achievement of the energy efficient buildings. However, very little data exist on the awareness of the persons that are supposed to incorporate these strategies in the design of buildings. The survey dataset contains the intent of promoting energy sustainability by architect while designing their building envelopes, the awareness of the building envelope strategies to adopt, factors influencing their adoption of these strategies, strategies that can be adopted to improve adoption. The dataset in this survey was obtained using questionnaires administered to both registered and non-registered architects in public housing organizations involved in the design, construction and management of selected housing estates in Lagos state. The questionnaire employed to determine the architect׳s perception on awareness and adoption of building envelope technologies (BET) for energy efficient housing (EEH) was based on a five-point Likert scale of variables selected from literature. The dataset include data from the seventy four (74) questionnaires that were returned out of the one hundred (100) questionnaires administered. Descriptive statistical tools of charts and tables were used to present the dataset. In Fig. 1, the breakdown of the registration status of architects showed that 29.4% of the architects were registered professionals with the regulatory council while 70.6% were unregistered architects. The industry working experience of the architects shown in Fig. 2 showed that the architects with less than 5 years working experience were only 59% of the respondents, while architects with 6 to 10 years were 13%, architects with 11 to 15 years were 10%, architects with 16 to 20 years were 10% and architects with over 20 years working experience in the construction industry were 8% of the total respondents. The dataset presented the knowledge based of the architects on the subject of building envelope technologies. In Fig. 3, the chart showed that 12% of the architects were highly knowledgeable on the use of building envelope technologies while 62% were knowledgeable, 16% were not sure on the use, 6% of the architects had a fair knowledge and 4% of the architects had no knowledge on the use of building envelope technologies. Fig. 4 revealed how often architects design with the intention to promote use of building envelope technologies in achieving energy efficient buildings. In Fig. 4, 7.8% always considered the use, while 25.5% often considered it, 35.3% sometimes considered it, 15.7% rarely considered it and 15.7% never considered the use of building envelope technologies in promoting energy efficient housing units. Fig. 5 showed the perception of the architects on the use of building envelope technologies will help reduce energy consumption in housing units in Lagos State. In Fig. 5, 43.1% of the architects believe it will always reduce the energy consumption, while 23.5% believe it sometimes will and 3.9% believe it rarely will help curtail the energy consumption in housing units in Lagos State. Table 1 showed the extent of architect׳s knowledge of using building envelope technologies in achieving energy efficient buildings. In Table 1, the building envelope strategies such as the use of Smart windows, Double-gazed windows, Advanced Insulations, Energy efficient HVAC, Photovoltaic for floors, Horizontal reflecting surfaces, Photovoltaic doors, External overhangs, Vacuum insulated wall panels, Photovoltaic windows, Energy efficient using LED lightning, Window attachments, Photovoltaic walls, Aerogel sealant for Air leakage, Photovoltaic foam for walls, Vegetation roofing and Photovoltaic roof were identified and ranked. Even though, the architects were aware about most of the building envelope technologies, the use of the system was different as shown in Table 2. Table 2 presented extent to which architects use building envelope technologies for efficient housing. In Table 2, Energy efficient using LED lightning, Window attachments, External overhangs and Photovoltaic roof were the most used strategies of building envelope technologies for energy efficient housing units. Table 3 showed the factors that influence the use of building envelope technologies for energy efficient housing. Factors such as Inadequate Knowledge, High aesthetics value, Good thermal comfort, Lack of established standard, Unwillingness to accept risks by architects, Low operating cost of building envelope technologies, Low impact on environment, Unwillingness to accept risks by clients, Concerns about privacy, Development control standards, Low aesthetics values, Time consuming to design with, Concerns about durability, Lack of material availability, Negative perception held by clients, Absence of construction guides and tools, Concerns about security, Lack of technical know-how, Site constraints, Low capital cost of building envelope technologies and Low energy consumption were identified and ranked. Table 4 highlighted suggested strategies to help increase the use of building envelope technologies for energy efficient housing. Table 4 ranked the strategies such as Obtaining more information on design policies and material performance, Inclusion of training programs on designing with building envelope technologies for energy efficient housing, Educating clients on the positives of being environmentally conscious, Reduction in technology costs and Seminars and lectures on the different types of building envelope technologies for energy efficient housing available and Reduction in material costs. The dataset is useful in developing countries where there is limitation in the power generation, transmission and distribution leading to limited power supply to housing units. It is pertinent for designers to consider the use of sustainable designs that engender energy efficiency through building designs and building materials specified. Designers should be less focused on aesthetics in housing schemes and more focused on providing environment friendly designs that meets the needs of the occupant and the environment.

**Table 1:** Extent of knowledge of using building envelope technologies (BET) to achieve energy efficient housing units.

**Awareness on Building Envelope Strategies**	**Highly aware**	**Aware**	**Undecided**	**Unaware**	**Highly unaware**	**SWV**	**INDEX**	**RANK**
Smart windows	24	39	3	4	4	297	4.0	1st
Double-gazed windows	21	39	6	3	5	290	3.9	2nd
Advanced Insulations	23	35	8	3	5	290	3.9	2nd
Energy efficient HVAC	19	43	4	2	6	289	3.9	2nd
Photovoltaic for floors	22	36	8	0	8	286	3.9	2nd
Horizontal reflecting surfaces	24	35	3	8	4	290	3.8	6th
Photovoltaic doors	23	31	7	10	3	283	3.8	6th
External overhangs	18	34	8	9	5	273	3.7	8th
Vacuum insulated wall panels	9	44	6	10	5	264	3.6	9th
Photovoltaic windows	12	39	9	9	5	266	3.6	9th
Energy efficient using LED lightning	13	38	9	9	5	267	3.6	9th
Window attachments	14	34	12	0	14	256	3.5	12th
Photovoltaic walls	11	37	12	10	4	263	3.5	12th
Aerogel sealant for Air leakage	22	29	5	4	5	254	3.4	14th
Photovoltaic foam for walls	14	27	10	13	10	244	3.3	15th
Vegetation roofing	14	27	10	13	10	244	3.3	15th
Photovoltaic roof	10	26	19	13	6	243	3.3	15th

**Table 2:** Perception on the use of building envelope technologies for energy efficient housing.

**Use of Building Envelope Technologies**	**Always**	**Often**	**Sometimes**	**Rarely**	**Never**	**SMV**	**INDEX**	**RANK**
Energy efficient using LED lightning	19	25	15	10	5	**265**	3.6	1st
Window attachments	14	25	21	9	5	256	3.5	2nd
External overhangs	14	25	18	14	4	256	3.5	2nd
Photovoltaic roof	15	29	10	8	12	249	3.3	4th
Energy efficient HVAC	8	26	18	12	10	232	3.1	5th
Double-gazed windows	3	16	25	20	10	204	2.7	6th
Aerogel sealant for Air leakage	4	10	25	16	19	186	2.5	7th
Horizontal reflecting surfaces	4	9	25	12	24	179	2.4	8th
Photovoltaic for floors	2	9	27	15	21	178	2.4	8th
Vacuum insulated wall panels	1	7	20	29	17	168	2.3	10th
Vegetation roofing	1	11	20	18	24	169	2.3	10th
Photovoltaic doors	2	12	14	22	24	168	2.3	10th
Smart windows	3	8	17	24	22	168	2.2	13th
Photovoltaic walls	4	6	10	26	28	154	2.1	14th
Photovoltaic windows	3	6	10	31	24	155	2.1	14th
Advanced Insulations	2	4	17	24	27	152	2.1	14th
Photovoltaic foam for walls	3	4	11	22	34	142	1.9	17th

**Table 3:** Factors influencing the use of building envelope technologies for energy efficient housing units.

**Factors**	**To a large extent**	**To some extent**	**Undecided**	**A little extent**	**Not at all**	**SMV**	**INDEX**	**RANK**
Inadequate Knowledge	41	22	0	6	5	310	4.2	1st
High aesthetics value	41	18	5	1	9	303	4.1	2nd
Good thermal comfort	19	43	4	2	19	302	4.1	2nd
Lack of established standard	29	29	8	3	5	300	4.1	2nd
Unwillingness to accept risks by architects	33	27	4	7	3	302	4.1	2nd
Low operating cost of building envelope technologies	19	25	15	10	5	265	3.8	6th
Low impact on environment	16	29	14	10	5	263	3.6	7th
Unwillingness to accept risks by clients	20	28	8	8	10	262	3.5	8th
Concerns about privacy	16	28	10	15	5	257	3.5	8th
Development control standards	15	29	9	17	4	256	3.5	8th
Low aesthetics values	15	28	13	6	12	250	3.4	11th
Time consuming to design with	12	30	14	11	7	251	3.4	11th
Concerns about durability	13	26	14	11	10	246	3.3	13th
Lack of material availability	6	39	9	13	7	246	3.3	13th
Negative perception held by clients	11	30	13	11	9	245	3.3	13th
Absence of construction guides and tools	8	28	16	12	10	234	3.2	16th
Concerns about security	12	20	19	15	8	235	3.2	16th
Lack of technical know how	9	26	19	9	11	235	3.2	16th
Site constraints	7	19	14	18	16	205	2.8	19th
Low capital cost of building envelope technologies	3	16	25	20	10	204	2.8	19th
Low energy consumption	8	7	15	36	8	193	2.6	21st

**Table 4:** Strategies to increase use of building envelope technologies for energy efficient housing.

**Strategies**	**Very important**	**Important**	**Neutral**	**Not important**	**Not very important**	**SMV**	**INDEX**	**RANK**
Obtaining more information on design policies and material performance	43	22	1	2	6	316	4.3	1st
Inclusion of training programs on designing with building envelope technologies for energy efficient housing	44	21	0	1	8	314	4.2	2nd
Educating clients on the positives of being environmentally conscious	41	23	0	4	6	311	4.2	2nd
Reduction in technology costs	36	28	2	2	6	308	4.2	2nd
Seminars and lectures on the different types of building envelope technologies for energy efficient housing available	42	21	1	1	9	308	4.2	2nd
Reduction in material costs	41	18	5	1	9	303	4.1	6^th^

## 2 Experimental design, materials and methods

Energy efficient housing is a type of building that implements solar architecture with modern technologies and energy efficient building materials to ensure that maximum comfort is attained with reduced energy cost without harm to the environment/climate [5]. The dataset was obtained using a cross-sectional survey method. The sampling method adopted for the study of the architects was multistage sampling technique. The procedure involved purposive selection of a city where architects are most concentrated leading to the choice of Lagos, and then random selection of architects within Lagos. Similar methods and contributions can be seen in [6], [7], [8], [9], [10], [11], [12], [13], [14], [15]. A total of seventy-four (74) registered and unregistered architects participated in the dataset. The dataset was collected in Lagos State. Lagos State was selected in this dataset due to its high population of over 20 million people with high need for housing units. Nigeria; a developing country at present generates a little above 5000 MW which is insufficient for its teeming population of over 200 million people in meeting its energy needs, therefore, the need for this dataset. A questionnaire instrument was used to obtain the dataset. The questionnaire was divided into five (5) sections. Using a 5-point Likert scale rating system for Section 2-4, adding all ratings for each isolate results in 15 points for overall user perception.

$$\mathrm{Thus};Q=\;\frac{\sum fx}{N}$$

Where, Q=Mean, Ʃ=Summation, Fx=Frequency of x and N=Number of occurrences. In order to obtain the perception aggregate index (I) of each service, a weight value of 5,4,3,2 and 1 was assigned to the ratings of the 5-point Likert scale. The summation of weight value (SWV) for each variable was obtained from the addition of the product of weight value of each rating and the number of responses of each rating. The perception aggregate index (I) for each variable was obtained from the division of each summation of value (SWV) by the total number of respondents which is represented as “N”.

$$\mathrm{Thus},\mathrm{Index}\left(I\right)=\frac{\mathrm{SWV}}{N}$$

By summing the nominal values and dividing by the total number of scaling variables, the cut-off point is determined. Dividing the total ratings of each variable gives a mean of 3. Thus, any mean above 3 indicates positive respondent׳s perception and below 3 indicates negative respondent׳s perception while a mean of exactly 3 shows neutral (undecided) on user perception by a respondent.

## References

[1] Sadineni S.B., Srikanth M., Boehm R.F. (2011). Passive building energy savings: a review of building envelope components. Renew. Sust. Energy Rev..

[2] Opoko A.P., Oluwatayo A.A., Ezema I.C., Opoko C.A. (2016). Residents׳ perception of housing quality in an informal settlement. Int. J. Appl. Eng. Res..

[3] A. Afolabi, M. Dada, An evaluation of the factors affecting housing and urban development projects in Lagos State. Paper presented at the Proceeding of CIB W107 Inter. Conference on Construction in developing countries and its contribution to sustainable development, 28th–30th January, 2014, University of Lagos, Lagos, Nigeria.

[4] Ibem E.O., Anosike M.N., Azuh D.E. (2011). Challenges in public housing provision in the post-independence era in Nigeria. Int. J. Hum. Sci..

[5] Nwofe P.A. (2014). Need for energy efficient buildings in Nigeria. Int. J. Energy Environ. Res..

[6] L. Amusan, P. Tunji-Olayeni, A. Afolabi, I. Omuh, R. Ojelabi, A. Oluwatobi, Remodularising technical institutions towards quality manpower delivery in construction sector in Nigeria, in: Proceedings of the 10th Annual International Technology, Education and Development Conference, 7th–9th March, 2016, Valencia, Spain.

[7] Tunji-Olayeni P., Emetere M.E., Afolabi A. (2017). Multilayer perceptron network model for construction material procurement in fast developing cities. Int. J. Civ. Eng. Technol..

[8] Oyeyipo O.O., Odusami K.T., Ojelabi R.A., Afolabi A.O. (2016). Factors affecting contractors׳ bidding decision for construction projects in Nigeria. J. Constr. Dev. Ctries..

[9] Okagbue H.I., Opanuga A.A., Oguntunde P.E., Ugwoke P.O. (2017). Random number datasets generated from statistical analysis of randomly sampled GSM recharge cards. Data Brief.

[10] Afolabi A., Fagbenle O.I., Mosaku T.O., Rocha Á. (2017). IT management of building materials׳ planning and control processes using web-based technologies.

[11] Popoola S.I., Atayero A.A., Badejo J.A., John T.M., Odukoya J.A., Omole D.O. (2018). Learning analytics for smart campus: data on academic performances of engineering undergraduates in Nigerian private university. Data Brief.

[12] Popoola S.I., Atayero A.A., Okanlawon T.T., Omopariola B.I., Takpor O.A. (2018). Smart campus: data on energy consumption in an ICT-driven university. Data Brief.

[13] Afolabi A.O., Ojelabi R.A., Tunji-Olayeni P.F., Fagbenle O.I., Mosaku T.O. (2018). Survey datasets on Women participation in Green jobs in the Construction Industry. Data Brief.

[14] Tunji-Olayeni P.F., Lawal P.O., Amusan L.M. (2012). Developing infrastructures in Nigeria: why is the cost so high?. Mediterr. J. Soc. Sci..

[15] Afolabi A.O., Ojelabi R.A., Bukola A., Akinola A., Afolabi A. (2018). Statistical exploration of dataset examining key indicators influencing housing and urban infrastructure investments in megacities. Data Brief.

