# Supplementary material for: Survey dataset on architect׳s awareness and adoption of building envelope technologies for energy efficient housing in Lagos State
Source: Data Brief. 2018 Jul 3;19:1894–901. doi: 10.1016/j.dib.2018.06.093 (PMC6141384; doi:10.1016/j.dib.2018.06.093)
Supplement: Supplementary file 2 — Supplementary material. [file mmc2.docx]

**
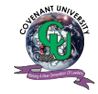
**

**DEPARTMENT OF ARCHITECTURE**

**COLLGE OF SCIENCE AND TECHNOLOGY**

**COVENANT UNIVERSITY, NIGERIA**

Dear Sir/ Madam

**PERCEPTION OF ARCHITECT’S IN THE USE OF BUILDING ENVELOPE TECHNOLOGIES FOR ENERGY EFFICIENCY IN LAGOS STATE**

Kindly give candid answers to the questions below. The questionnaire is designed to collect information for my research on investigating Architect’s perception in the use building envelope technologies for energy efficient buildings in Lagos state in view to improving energy efficiency in buildings .

Please be assured that the information provided by you will be treated in strict confidence and the results will be published only in an aggregated form. . I hope to give you a summary of the findings at the end of the research

Yours sincerely

Akinola Adedotun

dtn_akin@yahoo.com

**A SOCIO-ECONOMIC CHARACTERISTICS OF RESPONDENTS**

1. What is your gender? Male ( 1 ) female ( 2 )
2. How old are you? Below 30years ( 1 ), 31 -40 ( 2 ), 41-50 ( 3 ), 51-60 ( 4 ) 61-above (5 )
3. What is your marital status? Single ( 1 ) Married ( 2 ) Widowed ( 3 ) Divorced ( 4 )Separated ( 5 )
4. What is the highest level of education you have completed?

OND ( 1 ) HND ( 2 ) BSC/BTECH ( 3 ) MSC/BARCH ( 4 ) PHD ( 5 )

1. Are you a registered Architect? Yes ( 1 ) No( 2 )
2. If your answer in (5) is yes. When (Date )…………..
3. Organization

Private Architectural practice ( 1) Government Parastatals ( 2 ) Education ( 3 ) Manufacturing organization ( 4 ) Armed forces ( 5 )

1. What is your level of experience(years) <5 ( 1 ) 6-10 (2 ) 11-15 ( 3 ) 16-20 ( 4 ) >21(5 )
2. Monthly Income in Naira

20000 or less, (1) 21000- 70000, (2 ) 71,000- 120,000, ( 3 ) 121,000-170,000, (4 ) Above 170,000( 5 )

1. Can you say you have any knowledge of building envelope technologies for energy efficient housing? Not knowledgeable at all ( 1) Not knowledgeable (2 ) Not sure ( 3 )

Knowledgeable (4 ) Highly Knowledgeable (5 )

1. If yes, do you design/refurbish/build with the intention to promote building envelope technologies in energy efficient housing? Never( 1) Rarely(2 ) Sometimes(3 ) Often (4 ) Always (5)
2. Do you believe there is a need to promote building envelope technologies for energy efficient housing in Lagos state? Never(1 ) Rarely( 2 ) Sometimes(3 ) Often ( 4 ) Always (5 )
3. Do you think the use of building envelope technologies will reduce energy consumption in the State? Never( 1 ), Rarely(2), Sometimes(3), Often (4 ), Always ( 5 )

**B AWARENESS AND USE OF BUILDING ENVELOPE TECHNOLOGIES (BET) FOR ENERGY EFFICIENT HOUSING (EEH)**

To what extent are you aware that you can explore the following building envelope strategies to achieve passive energy Housing.

|  |  | Highly  unaware | Unaware | Undecided | Aware | Highly  aware |
| --- | --- | --- | --- | --- | --- | --- |
|  | Vacuum insulated wall panels | 1 | 2 | 3 | 4 | 5 |
|  | Double-glazed widows | 1 | 2 | 3 | 4 | 5 |
|  | Smart windows | 1 | 2 | 3 | 4 | 5 |
|  | Window attachments (e.g. shutters, shades, storm panel) | 1 | 2 | 3 | 4 | 5 |
|  | Advanced insulation (e.g. aerogel, VIPs) | 1 | 2 | 3 | 4 | 5 |
|  | Energy efficient HVAC | 1 | 2 | 3 | 4 | 5 |
|  | Energy efficient lighting using LED lighting | 1 | 2 | 3 | 4 | 5 |
|  | Photovoltaics Walls | 1 | 2 | 3 | 4 | 5 |
|  | Photovoltaics Windows | 1 | 2 | 3 | 4 | 5 |
|  | Photovoltaics roofs | 1 | 2 | 3 | 4 | 5 |
|  | Photovoltaics doors | 1 | 2 | 3 | 4 | 5 |
|  | Polyurethane foam for the roof(insulation) | 1 | 2 | 3 | 4 | 5 |
|  | Polyurethane foam for walls(insulation) | 1 | 2 | 3 | 4 | 5 |
|  | Polystyrene for floors. | 1 | 2 | 3 | 4 | 5 |
|  | Aerogel sealants for Air leakage | 1 | 2 | 3 | 4 | 5 |
|  | External overhangs (fins) | 1 | 2 | 3 | 4 | 5 |
|  | Horizontal reflecting surfaces (light shelves | 1 | 2 | 3 | 4 | 5 |
|  | Vegetation roofing | 1 | 2 | 3 | 4 | 5 |

To what extent do you use the following building envelope technologies for energy efficient housing

|  |  | Never | Rarely | Sometimes | often | Always |
| --- | --- | --- | --- | --- | --- | --- |
|  | Vacuum insulated wall panels | 1 | 2 | 3 | 4 | 5 |
|  | Double-glazed | 1 | 2 | 3 | 4 | 5 |
|  | Smart windows | 1 | 2 | 3 | 4 | 5 |
|  | Window attachments (e.g. shutters, shades, storm panel) | 1 | 2 | 3 | 4 | 5 |
|  | Advanced insulation (e.g. aerogel, VIPs) | 1 | 2 | 3 | 4 | 5 |
|  | Energy efficient HVAC | 1 | 2 | 3 | 4 | 5 |
|  | Energy efficient lighting using LED lighting | 1 | 2 | 3 | 4 | 5 |
|  | Photovoltaics Walls | 1 | 2 | 3 | 4 | 5 |
|  | Photovoltaics Windows | 1 | 2 | 3 | 4 | 5 |
|  | Photovoltaics roofs | 1 | 2 | 3 | 4 | 5 |
|  | Photovoltaics doors | 1 | 2 | 3 | 4 | 5 |
|  | Polyurethane foam for the roof(insulation) | 1 | 2 | 3 | 4 | 5 |
|  | Polyurethane foam for walls(insulation) | 1 | 2 | 3 | 4 | 5 |
|  | Polystyrene for floors. | 1 | 2 | 3 | 4 | 5 |
|  | Air leakage by using aerogel sealants | 1 | 2 | 3 | 4 | 5 |
|  | External overhangs (fins) | 1 | 2 | 3 | 4 | 5 |
|  | Horizontal reflecting surfaces (light shelves | 1 | 2 | 3 | 4 | 5 |
|  | Vegetation roofing | 1 | 2 | 3 | 4 | 5 |
|  | Solar hot-tap water system | 1 | 2 | 3 | 4 | 5 |

**C. FACTORS THAT INFLUENCE THE USE OF BUILDING ENVELOPE TECHNOLOGIES (BET) FOR PASSIVE ENERGY HOUSING (PEH)**

**To what extent do the following influence the building envelope strategies listed in Section B**

**Tick as appropriate**

|  | Factors | Not at all | A little extent | Undecided | To some extent | To a large extent |
| --- | --- | --- | --- | --- | --- | --- |
|  | Inadequate Knowledge | 1 | 2 | 3 | 4 | 5 |
|  | Lack of material Availability | 1 | 2 | 3 | 4 | 5 |
|  | Site constraints | 1 | 2 | 3 | 4 | 5 |
|  | Lack of established standard/policy | 1 | 2 | 3 | 4 | 5 |
|  | Low aesthetics value | 1 | 2 | 3 | 4 | 5 |
|  | Unwillingness to accept risks by clients | 1 | 2 | 3 | 4 | 5 |
|  | Unwillingness to accept risks by the architect | 1 | 2 | 3 | 4 | 5 |
|  | Negative perception held by clients | 1 | 2 | 3 | 4 | 5 |
|  | Lack of technical know-how | 1 | 2 | 3 | 4 | 5 |
|  | Development control standards | 1 | 2 | 3 | 4 | 5 |
|  | Concerns about Privacy | 1 | 2 | 3 | 4 | 5 |
|  | Concerns about Durability | 1 | 2 | 3 | 4 | 5 |
|  | Concerns about security | 1 | 2 | 3 | 4 | 5 |
|  | Time consuming to design with | 1 | 2 | 3 | 4 | 5 |
|  | Absence of construction guides and tools | 1 | 2 | 3 | 4 | 5 |
|  | Low energy consumption | 1 | 2 | 3 | 4 | 5 |
|  | Low impact on the environment | 1 | 2 | 3 | 4 | 5 |
|  | Aesthetics | 1 | 2 | 3 | 4 | 5 |
|  | Low operating cost of the building envelope technologies | 1 | 2 | 3 | 4 | 5 |
|  | Good thermal comfort | 1 | 2 | 3 | 4 | 5 |
|  | Low capital cost of the building envelope technologies | 1 | 2 | 3 | 4 | 5 |
|  | Solar hot-tap water system | 1 | 2 | 3 | 4 | 5 |

**D. POSSIBLE SUGGESTIONS FOR INCREASED USE OF BUILDING ENVELOPE TECHNOLOGIES (BET) FOR ENERGY EFFICIENT HOUSING ( EEH)**

What ways can the use of building envelope technologies be enhanced? Please tick as appropriate

|  | Solutions | Very unimportant | somewhat unimportant | Undecided | Somewhat Important | Extremely Important |
| --- | --- | --- | --- | --- | --- | --- |
|  | Reduction in technologies cost | 1 | 2 | 3 | 4 | 5 |
|  | Obtaining more information on design policies and material performance | 1 | 2 | 3 | 4 | 5 |
|  | Inclusion of training programs on designing with BET FOR EEH | 1 | 2 | 3 | 4 | 5 |
|  | Educating clients on the positives of being environmentally conscious | 1 | 2 | 3 | 4 | 5 |
|  | Seminars and lectures on the different types of BET for EEH Available and the benefits of using them | 1 | 2 | 3 | 4 | 5 |

Please give other reasons on the use building envelope technologies for energy efficiency not covered here?

……………………………………………………………………………………………………………………………………………………………………………………………………………………………………………………………………………………………………………………………………………………………………………………………………………………………………………………………………………………………………………………………………………………………………………………………………………………………………………………………………………………………………………………………………………………….

Please list other building envelope technologies for energy efficiency not covered here?

……………………………………………………………………………………………………………………………………………………………………………………………………………………………………………………………………………………………………………………………………………………………………………………………………………………………………………………………………………………………………………………………………………………………………………………………………………………………………………………………………………………………………………………………………………………………………………………………………………………………………………………………………………………………………………………………………………………..
